# Artificial Visual System for Stereo-Orientation Recognition Based on Hubel-Wiesel Model

**DOI:** 10.3390/biomimetics10010038

**Published:** 2025-01-08

**Authors:** Bin Li, Yuki Todo, Zheng Tang

**Affiliations:** 1 Division of Electrical Engineering and Computer Science, Kanazawa University, Kanazawa-shi 920-1192, Japan; crislee@stu.kanazawa-u.ac.jp; 2 Faculty of Electrical, Information and Communication Engineering, Kanazawa University, Kanazawa-shi 920-1192, Japan; 3 Institute of AI for Industries, Chinese Academy of Sciences, 168 Tianquan Road, Nanjing 211100, China; 4 School of Computer Engineering and Science, Shanghai University, Shanghai 200444, China

**Keywords:** stereo-orientation selectivity, Hubel-Wiesel model, artificial visual system

## Abstract

Stereo-orientation selectivity is a fundamental neural mechanism in the brain that plays a crucial role in perception. However, due to the recognition process of high-dimensional spatial information commonly occurring in high-order cortex, we still know little about the mechanisms underlying stereo-orientation selectivity and lack a modeling strategy. A classical explanation for the mechanism of two-dimensional orientation selectivity within the primary visual cortex is based on the Hubel-Wiesel model, a cascading neural connection structure. The local-to-global information aggregation thought within the Hubel-Wiesel model not only contributed to neurophysiology but also inspired the development of computer vision fields. In this paper, we provide a clear and efficient conceptual understanding of stereo-orientation selectivity and propose a quantitative explanation for its generation based on the thought of local-to-global information aggregation within the Hubel-Wiesel model and develop an artificial visual system (AVS) for stereo-orientation recognition. Our approach involves modeling depth selective cells to receive depth information, simple stereo-orientation selective cells for combining distinct depth information inputs to generate various local stereo-orientation selectivity, and complex stereo-orientation selective cells responsible for integrating the same local information to generate global stereo-orientation selectivity. Simulation results demonstrate that our AVS is effective in stereo-orientation recognition and robust against spatial noise jitters. AVS achieved an overall over 90% accuracy on noise data in orientation recognition tasks, significantly outperforming deep models. In addition, the AVS contributes to enhancing deep models’ performance, robustness, and stability in 3D object recognition tasks. Notably, AVS enhanced the TransNeXt model in improving its overall performance from 73.1% to 97.2% on the 3D-MNIST dataset and from 56.1% to 86.4% on the 3D-Fashion-MNIST dataset. Our explanation for the generation of stereo-orientation selectivity offers a reliable, explainable, and robust approach for extracting spatial features and provides a straightforward modeling method for neural computation research.

## 1. Introduction

Human visual perception of the external environment relies on various fundamental feature recognition processes, including motion, color, and orientation [1,2]. These primary visual feature extractions predominantly occur in the primary visual cortex and are subsequently integrated into the higher-level cortex for abstract feature recognition [3,4]. Among these fundamental visual features, orientation feature response plays a crucial role in object edge detection, texture analysis, and shape recognition [5,6]. The neural mechanism of selective response to stimuli with preferred orientation is referred to as orientation selectivity [7]. Given this neuron property, many researchers conducted neural modeling based on orientation selectivity and inspired the development of artificial visual systems toward two-dimensional vision tasks [8,9,10]. As autonomous driving and robotics continue to advance, there is an increasing demand for processing three-dimensional spatial information inputs [11,12]. Therefore, investigating and modeling the neural mechanisms of spatial information recognition is beneficial for implementing robust information processing systems and advancing computational neuroscience.

The most remarkable study on orientation selectivity was conducted by Hubel and Wiesel, who observed several types of cortical neurons in the mammalian primary visual cortex exhibiting selective responses to visual stimuli [13,14]. They employed the terms V1 simple cells and complex cells to describe two typical neuron types with orientation selectivity [15,16]. Simple cells have relatively small receptive fields and show a preference for specific stimulus patterns [14]. These cells are vigorously activated only by stimuli with a particular orientation within their receptive field, while they exhibit no or weaker responses to other orientations [15]. On the other hand, complex cells also demonstrate selectivity towards stimulus orientation but possess larger receptive fields [15]. Unlike simple cells, complex cells maintain their response when preferred orientation stimuli move within their receptive field [14]. Hubel and Wiesel suggested that the generation of orientation selectivity originates from the hierarchical structure between LGN (lateral geniculate nucleus) and V1 (primary visual cortex) [17,18]. Specifically, V1 simple cells receive inputs from multiple LGN cells whose spatially close receptive fields are organized in a specific orientation pattern [3,19]. This arrangement leads to the development of corresponding orientation selectivity in simple cells. The complex cell is connected with those simple cells with spatially close receptive fields that share the same preferred orientation, resulting in a larger receptive field for complex cell responses while maintaining their selectivity to preferred stimuli orientations [17,20]. This explanation for the generation structure of orientation selectivity is commonly referred to as the Hubel-Wiesel model, which is extensively utilized in artificial visual system modeling [3,8,9,10,21]. The Hubel-Wiesel model is a concise and generalized concept that explains the feature extraction and aggregation process within the brain. Its thought of cascade connection and local-to-glocal information aggregation propelled the development of computational neuroscience [3,22,23,24,25,26] and computer vision [9,10,27,28,29,30].

The recognition of 3D information primarily occurs in the high-level visual cortical area [3,31,32]. Within these higher-order cortical areas, numerous neurons exhibit sensitivity to various 3D information, such as spatial depth, shape, curvature, surface characteristics, 3D fragments, and disparity [33,34,35,36,37,38,39,40]. While some researchers have reported that certain V4 neurons display stereo-orientation tuning without additional depth cues, it is more commonly observed that a substantial number of neurons exhibit cooperative responses to distinct spatial information [35,39,41]. The complexity associated with spatial information poses challenges in modeling spatial feature selectivity. However, it is worth noting that, similar to the generation process of two-dimensional orientation selectivity described in the Hubel-Wiesel model, the feature selectivity for spatial information in high-order cortical neurons also arises from the convergence of low-level simple features [8,19,42,43,44,45,46]. This cascading property offers a potential method for quantitatively reconstructing the neural mechanism of spatial feature selectivity [46,47,48].

Current research on stereo-orientation selectivity still focuses on discovering and observing the neuron activation or simulating the neuron activation and neural circuit connection [46,49,50,51,52]. There are limited attempts to develop a concise modeling scheme and further explore its application in stereo-orientation recognition. In addition, prior orientation recognition-related work mainly focuses on two-dimensional orientation, and detection techs include deep learning methods and traditional feature-matching methods [53,54,55,56,57,58,59]. Traditional techniques commonly have high computational complexity and poor adaptability to complex scenes [60]. Accordingly, the deep learning method is gradually being widely employed for object orientation detection tasks. The deep learning methods could obtain good performance after training with large amounts of data but require high computing resources and also lack robustness to adverse environmental conditions [30].

Enhancing the performance of systems in adverse environmental conditions is also a significant research topic [26,61,62]. Techniques for this issue include image preprocessing and enhancement [62,63,64,65], domain adaptation and transfer learning [66,67], adversarial training and self-supervised learning [68,69], and multimodal fusion [70,71]. In addition, biomimetic approaches also demonstrate significant potential in improving system performance [72,73]. For instance, brain cognitive computing can be employed to optimize the prediction of the flow state of the rectifier [74], using the Kolmogorov-Arnold network to predictive model electrohydrodynamic pumping (a biomimetic system) with interpretability [75], and biological visual mechanism can be applied to construct robust and explainable bio-inspired networks [76]. Especially in the image recognition task, introducing the biological visual mechanism into the construction of deep neural networks is also demonstrated as an efficient method to improve deep models’ robustness and overall performance [30,76,77]. However, most bio-inspired neural networks concentrated on two-dimensional vision and image task, there is a lack of application and innovation in three-dimensional tasks. On one hand, spatial information differs from image information, and traditional Gabor filter-based methods are unsuitable for 3D tasks. On the other hand, we still know little about the recognition mechanism of spatial information within high-order visual cortical layers.

To extend the application of feature selectivity neuron properties to three-dimensional tasks, we propose a concise but efficient modeling strategy for stereo-orientation selectivity based on local-to-global information aggregation within the cascade Hubel-Wiesel model and develop an artificial visual system (AVS) for three-dimensional recognition tasks. We suggest initially employing depth selective cells to extract diverse depth information. Subsequently, the various depth information extracted by depth selective cells is combined in simple stereo-orientation selective cells to generate different local stereo-orientation selectivity. Lastly, the outputs from the simple stereo-orientation selective cells with identical stereo-orientation selectivity are integrated into a complex stereo-orientation selective cell, resulting in global stereo-orientation selectivity. Extensive simulation results demonstrate that our proposed mechanism of stereo-orientation selectivity is reliable, while the AVS based on this mechanism proves effective and stable in recognizing orientation information. Furthermore, compared to extensive deep neural network methods, our AVS exhibits considerable superiority in robustness against spatial noise jitters. The contributions of this paper are:

- We proposed a straightforward and concise modeling strategy for the generation of stereo-orientation selectivity and developed an AVS for stereo-orientation selectivity.
- The AVS is demonstrated with robustness, superiority, and efficiency in object oreintation detection tasks compared with deep models.
- Implementing AVS connected with deep models helps enhance deep models’s performance, robustness, and stability in 3D object classification tasks.
- Our work provides a robust spatial feature pre-extraction method for 3D object recognition tasks and a potential explanation of how the brain perceives and encodes stereo-orientation information.

## 2. Methods

In this section, we introduce the mechanism of stereo-orientation selectivity and details of the artificial visual system (AVS) for stereo-orientation recognition. Firstly, we provide explanations regarding the generation of stereo-orientation selectivity based on the Hubel-Wiesel model. Subsequently, we give the modeling method of simple cells and complex cells. Lastly, we describe the implementation process of AVS.

### 2.1. Hubel-Wiesel Model and Stereo-Orientation Selectivity

**Figure 1:**
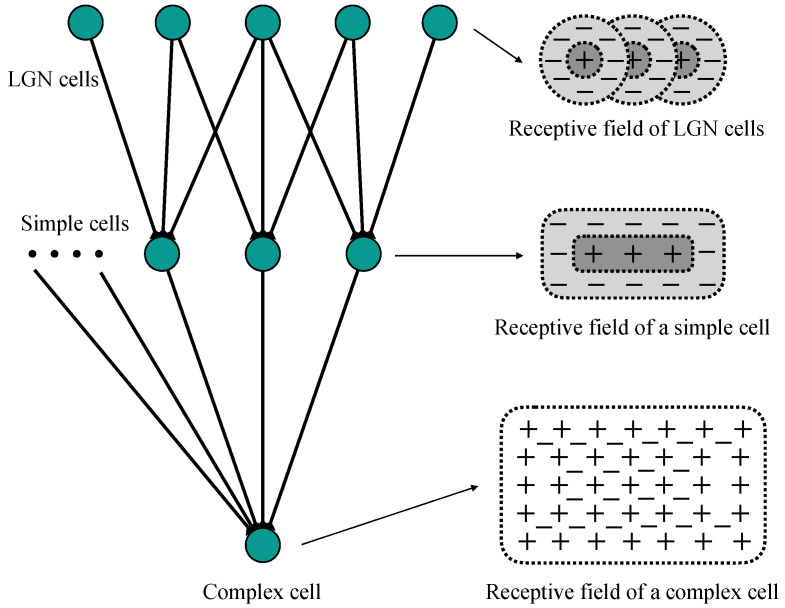
The hierarchical cascade structure of the Hubel-Wiesel model.

The Hubel-Wiesel model describes the primary visual pathway as a cascade structure to explain the generation of orientation selectivity. Figure 1 illustrates the structure of the Hubel-Wiesel model. This model exhibits a hierarchical cascade architecture, wherein simple cells process local orientation information while complex cells integrate outputs from simple cells to encode more complex features. Specifically, the simple cell receives input from the LGN cells, whose receptive fields are arranged in a specific way that makes the simple cell sensitive to stimuli with specific orientations. Complex cell integrates inputs from multiple simple cells within an extensive receptive field to generate global orientation selectivity.

**Figure 2:**
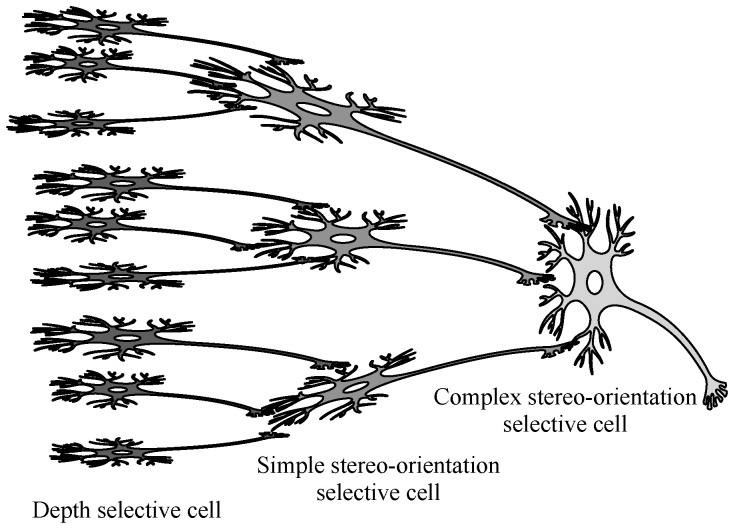
The conceptually neural connections of stereo-orientation selectivity.

Previous studies have indicated that spatial features such as shape and curvature are derived from the integration of distinct depth information [78,79,80]. Drawing inspiration from the hierarchical cascade structure of the Hubel-Wiesel model, which emphasizes sequential processing and information integration, we propose an explanation for the generation of stereo-orientation selectivity. We suggest integrating multiple depth cues arranged in a specific spatial configuration to obtain stereo-orientation perception. Specifically, we initially employ depth selective cells to extract various positional information related to depth. Subsequently, each type of simple stereo-orientation selective cell receives inputs from several depth selective cells whose extracted depth information can be organized into a specific orientation within local space, thereby generating local stereo-orientation selectivity. Lastly, all simple stereo-orientation selective cells with the same orientation preference output to a complex stereo-orientation selective cell, resulting in the generation of global orientation selectivity. The conceptually neural connection underlying stereo-orientation selectivity is presented in Figure 2.

### 2.2. Simple Stereo-Orientation Selective Cell

**Figure 3:**
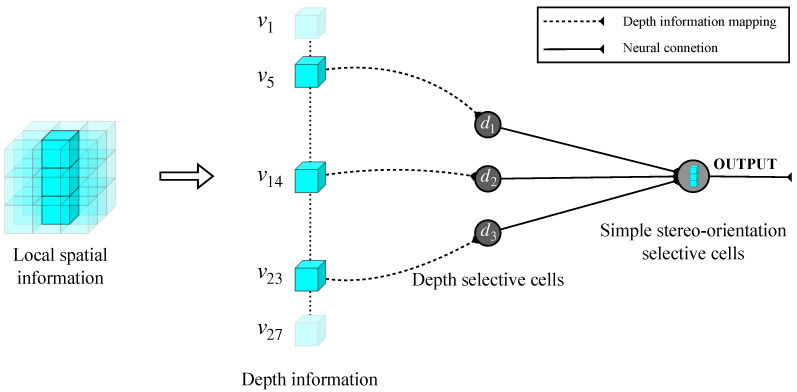
The information processing flow for local stereo-orientation information.

Local orientation selectivity is generated by simple stereo-orientation selective cells through the integration of specific depth information within a defined local space, which is extracted by depth selective cells. To quantify the computation of stereo-orientation selectivity, a fixed-scale local space with dimensions of 3×3×3 is designed, allowing for the definition of 13 specific stereo-orientations within each local space. Figure 3 illustrates an instance of the information processing flow for local stereo-orientation information.

The depth selective cells do not directly accept the spatial information and are commonly reported as existing in high-order cortical layers [44,81,82,83]. There are multiple middle stages before depth selective cells respond to preferred depth information. However, the presence of depth information will truly activate specific depth selective cells, establishing a definitive causal relationship [3,82,83]. Therefore, the recognition process of spatial depth information can be directly simplified by mapping the voxel value of spatial elements at specific positions within a local space as direct inputs to depth selective cells. Within a local voxel space sized as 3×3×3, 27 spatial elements can be identified and denoted from $v_{1}$ to $v_{27}$. Accordingly, the activation *d* of a depth selective cell can be quantitatively defined as follows:

(1) $$d=v_{index}\;,v_{index}=\left\{\begin{array}{cc} 1 & ,\;\mathrm{if}\;\mathrm{spatial}\;\mathrm{element}\;\mathrm{existed};\\ 0 & ,\mathrm{otherwise}. \end{array}\right.$$

$v_{index}$ denotes the voxel value (0 or 1) of a specific spatial element.

**Figure 4:**
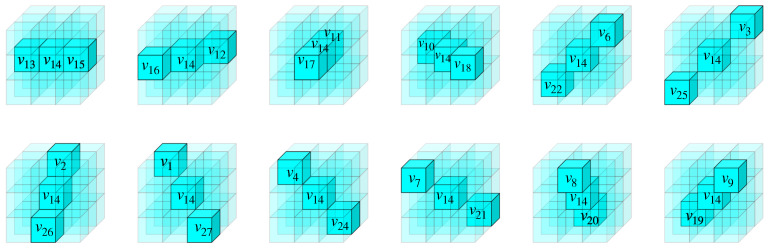
The remaining 12 types of stereo-orientation and their element indices within a local space.

The simple stereo-orientation selective cells receive inputs from three specific depth selective cells, which provide corresponding depth information that can be combined to form a local stereo-orientation feature. As illustrated in Figure 3, the three depth selective cells extract distinct depth information, which collectively contributes to a vertical orientation feature. Subsequently, the simple stereo-orientation selective cell integrates these inputs and exhibits selectivity towards vertical orientation. We employed the Sigmoid function to model simple cell activation, a widely employed technique in the field of neural modeling [84,85,86]. The activation value *s* of a simple stereo-orientation selective cell is defined by the following formula:

(2) $$s=\frac{1}{1+e^{-k(\sum d_{i}-\theta )}}\;,$$

where *k* denotes the response sensitivity of the cell, default as $10^{4}$. $d_{i}$ represents the inputs of each depth selective cell, and θ is the activation threshold, set to 2.5. We designed 13 distinct types of simple stereo-orientation selective cells, each corresponding to a specific local stereo-orientation defined within a local space. With the exception of the vertical orientation illustrated in Figure 3, the remaining 12 types of local stereo-orientation are depicted in Figure 4.

### 2.3. Complex Stereo-Orientation Selective Cell

**Figure 5:**
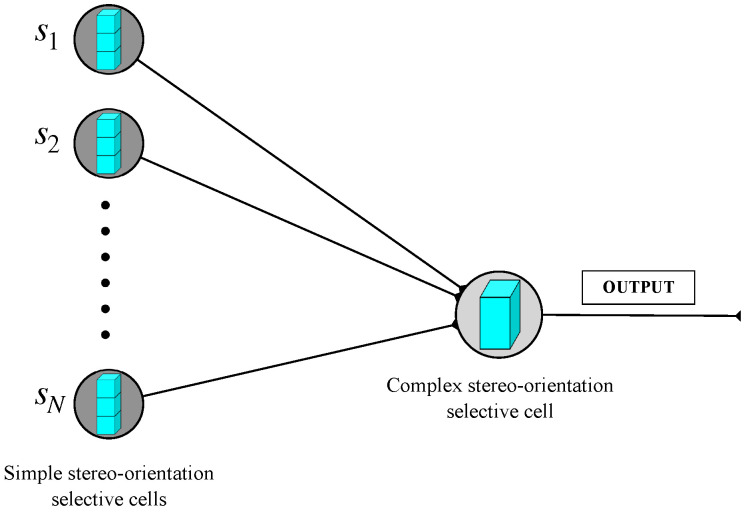
The connection pattern between simple and complex stereo-orientation selective cell.

Based on our explanation for the mechanism of stereo-orientation selectivity, the complex stereo-orientation selective cells are responsible for the integration of local stereo information extracted by simple stereo-orientation selective cells. The connection pattern between simple and complex stereo-orientation selective cells is illustrated in Figure 5. We define the response rule of complex stereo-orientation selective cells as follows:

(3) $$c=\sum_{i=1}^{N}s_{i}\;,$$

where *c* denotes the activation value of complex stereo-orientation selective cell, and *N* represents the total number of simple stereo-orientation selective cells. $s_{i}$ denotes the inputs of each simple stereo-orientation selective cell.

### 2.4. Artificial Visual System for Stereo-Orientation Recognition

**Figure 6:**
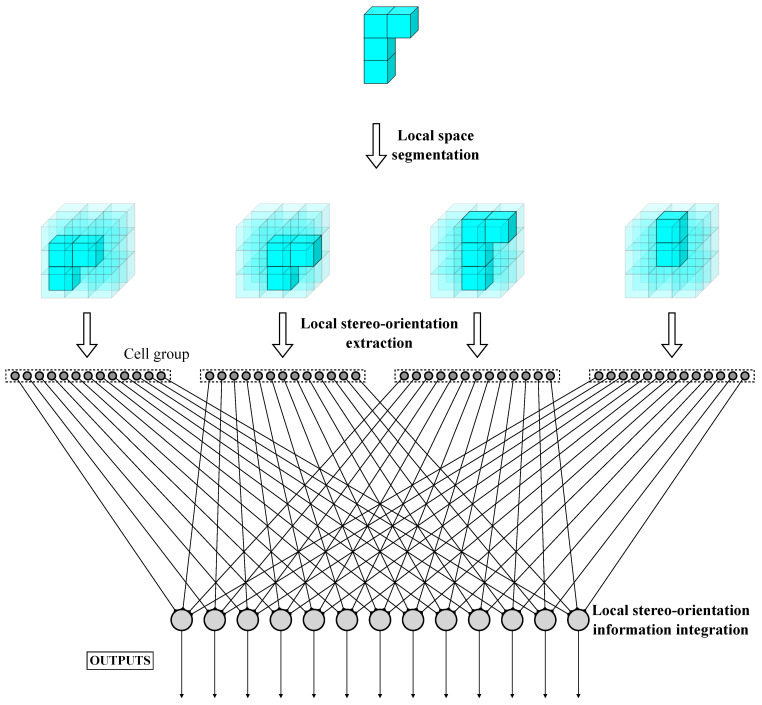
The overall process flow of AVS for stereo-orientation recognition.

We consider that spatial information is organized in a similar way as image information. The global feature is the combination and connection of local feature fragments, and each fragment can be continually separated into multiple dependent spatial elements. Inversely, we can extract all local feature fragments by recognizing specific spatial elements and subsequently obtain the global features. Based on such thought of local-to-global information aggregation, we implemented an artificial visual system (AVS) for stereo-orientation recognition tasks. The overall process flow of AVS for object orientation recognition is depicted in Figure 6. For the object, we propose first taking each element of the object as the central point to segment out multiple local spaces. Subsequently, we employ a group of simple stereo-orientation selective cells consisting of 13 types that correspond to the defined local stereo-orientations within each local space to recognize the local stereo-orientation. Finally, these simple stereo-orientation selective cells are connected to complex stereo-orientation selective cells with identical stereo-orientation selectivity. The complex stereo-orientation selective cells integrate inputs from each cell group and serve as the output of AVS. The complex stereo-orientation selective cell with the greatest activation value indicates global object stereo-orientation.

## 3. Simulations and Results

### 3.1. Implementation Details

We mainly conducted three experiments based on the artificial visual system (AVS): Physiological visual simulation, stereo-orientation recognition, and 3D object classification. In this subsection, we give the implementation details of all experiments, including platform, implemented codes, evaluation criteria, and datasets.

Physiological visual simulation:

- Research question Validating the orientation selectivity of AVS
- Dataset 1 A grating dataset comprises 1600 frames, including 800 frames featuring gratings with four specific orientations and eight orthogonal moving directions, and 800 frames of blank data. Each 100 frames of drift motion was followed by 100 frames of static blank window simulating the resting duration after drifting.
- Dataset 2 A random dot dataset comprised 1300 3D data, and each consisted of 300 randomly positioned dots. Based on the original random dot dataset, randomly select several dots and perform specific forward motion and reverse motion to generate local orientation information. Through this process, five additional random dot datasets with local orientation fragments (number of moving dots: 1, 2, 3, 4, 5) were generated.
- Evaluation criteria The response intensity of complex stereo-orientation selective cells during grating drifting.

Stereo-orientation recognition

- Research question Validating the effectiveness of AVS in stereo-orientation information extraction and the superiority compared with deep models.
- Dataset An artificial object orientation dataset consists of various objects with specific orientations and scales (line segment or bar). Multiple noise test sets with varying intensities of background noise. Training set size: 15,600; Validation set size: 5200; Test set size: 5200.
- Evaluation criteria Detection accuracy on clean and noise stereo-orientation data.

3D object classification

- Research question Validating the effectiveness of AVS in enhancing deep model’s robustness against noise.
- Dataset 3D-MNIST and 3D-Fashion-MNIST datasets derived from the original MNIST [10] and Fashion-MNIST image dataset [87].
- Evaluation criteria Detection accuracy on clean and noise 3D object datasets and the standard deviation of repeated simulations.

Abaltion study

- Research question Validating the contribution of each component in AVS to deep model performance enhancement.
- Dataset 3D-MNIST datasets.
- Evaluation criteria Detection accuracy of the deep model with different feature selection methods on clean 3D object datasets.

All experiments were implemented on the Apple M1 Max chips (Apple Inc., Cupertino, CA, USA) and NVIDIA GeForce RTX 3090 hardware environment (NVIDIA Corporation, Santa Clara, CA, USA). Implementation code related to this paper is available from the corresponding author upon request.

## 3.2. Physiological Visual Simulation

**Figure 7:**
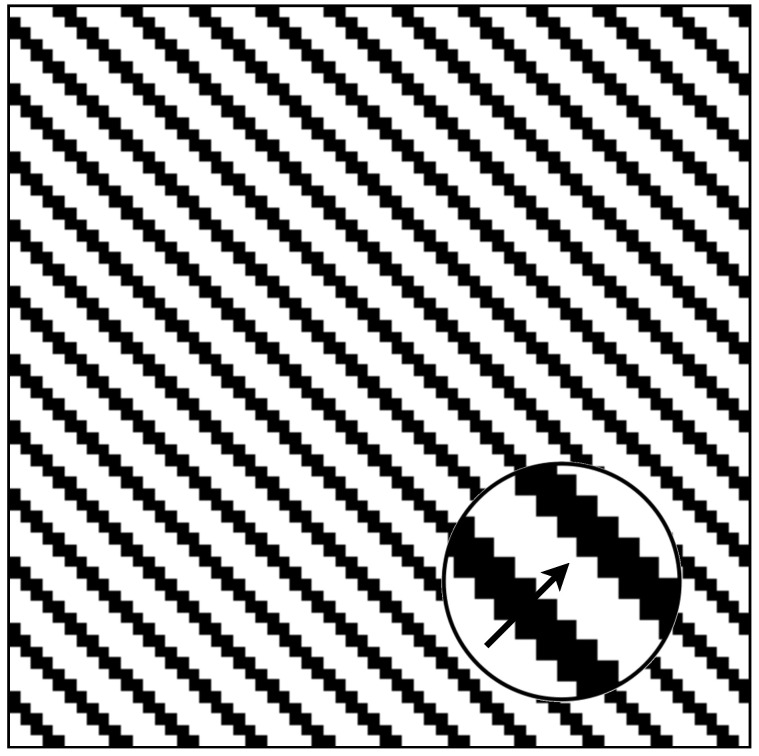
One frame of drift grating stimuli data.

In the field of orientation selectivity research, drift gratings are commonly employed to stimulate visual neurons and record their responses, thereby determining their orientation selectivity. Similarly, we utilized drift gratings to initially validate the orientation selectivity of the artificial visual system (AVS) in our study. Figure 7 illustrates one frame of the drift grating data.

**Figure 8:**
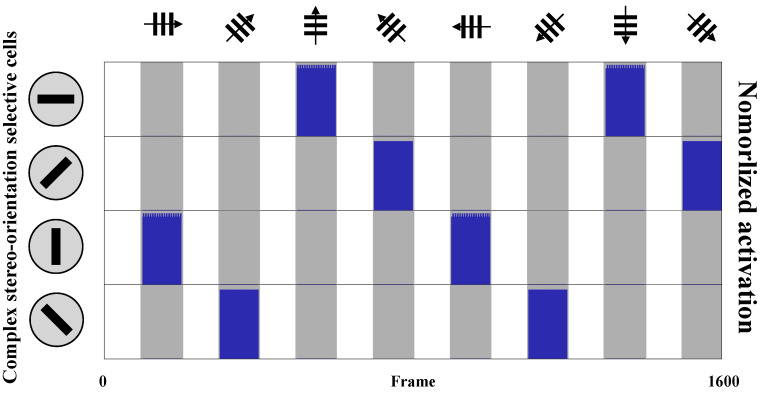
Activation records of complex stereo-orientation selective cells($0^{\circ }$, $45^{\circ }$, $90^{\circ }$, and $135^{\circ }$). The white area represents the rest state of stimuli, the gray area represents the moving state of stimuli, and the blue color denotes the neuron activation.

We employed AVS to detect the global orientation ($0^{\circ }$, $45^{\circ }$, $90^{\circ }$, or $135^{\circ }$) within each frame of drift grating data and recorded the responses of complex stereo-orientation selective cells. The normalized activation outputs of four corresponding complex stereo-orientation selective cells are presented in Figure 8. As expected, for each specific orientation of the drift grating data, the respective selective cells exhibited vigorous activation throughout the entire motion period, while remaining in a resting state for non-preferred orientation. The simulation records are consistent with the previous results of physiological research [13,19,88] and initially demonstrate the feasibility of AVS in object orientation recognition.

**Figure 9:**
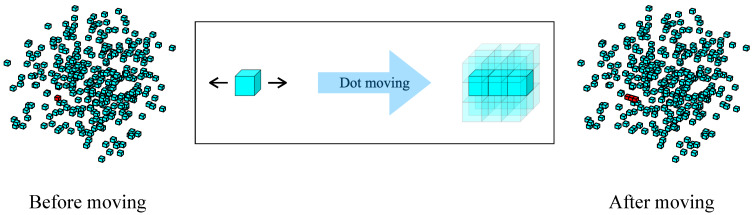
The generation process of oreintation information in random dots.

Moreover, the random dot pattern is also widely utilized for validating neuron activation in the field of stereovision research [89]. A random dot pattern consists of a large number of points that are spatially randomly distributed without any clear features. This allows the study of neuron responses to be as free from other cues as possible, thus enabling a pure investigation of the visual system’s response to specific attributes. In this research, we select several dots in each 3D random dot data to perform specific forward and reverse motions and generate local stereo-orientation information for each data, as illustrated in Figure 9. Subsequently, we evaluated the AVS’s sensitivity to oreintation information fragments within a large amount of random dots.

**Figure 10:**
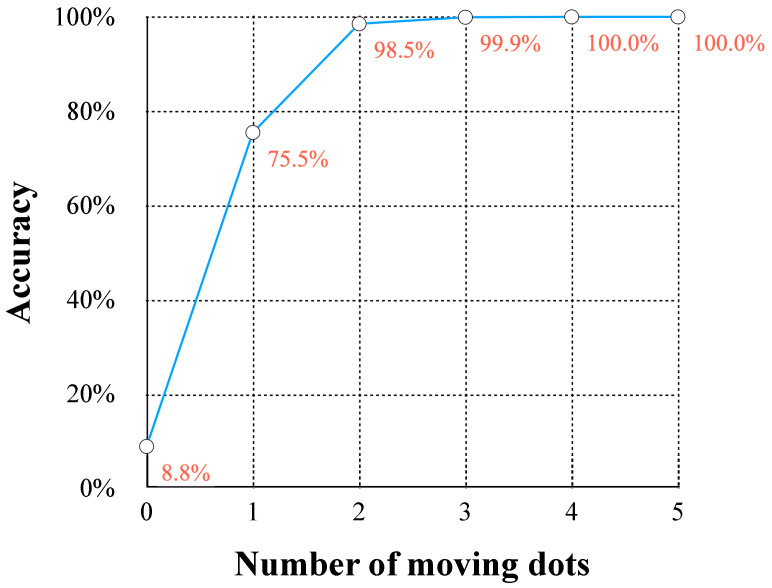
Acuuracy curve of AVS across different random dot datasets.

Summarized the detection accuracy of AVS on random dots datasets with different numbers of moving dots and illustrated in Figure 10. When the number of moving dots is zero, there is no orientation information, and the accuracy rate of AVS is 8.8%. Notably, even a single moving dot can provide effective orientation information for AVS (with 75.5% accuracy), and when the number of moving dots increased to three, AVS could achieve almost perfect performance. The results on the random dot pattern demonstrated that AVS is effective and robust in extracting orientation features under complex information disturbance.

## 3.3. Stereo-Orientation Recognition

**Figure 11:**
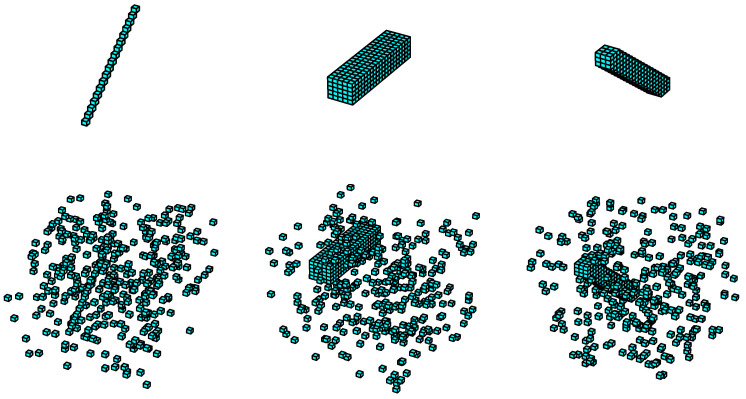
Instances of clean data and noise data.

The effectiveness and robustness of AVS in stereo-orientation recognition are evaluated on the artificial object orientation datasets. Figure 11 presents several instances of both clean data and noise data.

The robustness of AVS against spatial noise jitter is evaluated using these noise test sets. We also employed multiple deep neural network models for object orientation recognition tasks. The voxel-based models were pre-loaded with ImageNet-based pre-trained parameters, and the point cloud-based models were trained without pre-trained parameters (see Table A1 in Appendix A for more details of training settings). The performances of various models are presented in Table 1.

**Accuracy under Different Noise Intensities** In the orientation detection task, all deep models exhibited good performance on clean data. The TransNeXt and PointMLP models even realized 100% accuracy. However, these deep models performed poorly under noise disturbance, and the accuracy dropped significantly as noise intensity increased. In contrast, AVS not only achieved perfect performance on clean data (100% accuracy), but it also demonstrated remarkable robustness to noise. AVS maintains 92.73% accuracy even at 5% intensity of noise.

**Model Complexity** ConvNeXt and UniRepLKNet models possess the most parameters but did not attain an expected model performance. And despite the PointMLP model’s highest FLOPs of 7890 M, its robustness to noise was virtually none. In contrast, AVS achieved excellent performance with only 353 parameters and FLOPs of 9.48 M.

In short, deep models require high computing resources but perform poorly on noise data, while the AVS achieves the optimal performance with the lowest computational cost. AVS is feasible, efficient, and robust in stereo-orientation information extraction.

## 3.4. 3D Object Classification

**Figure 12:**
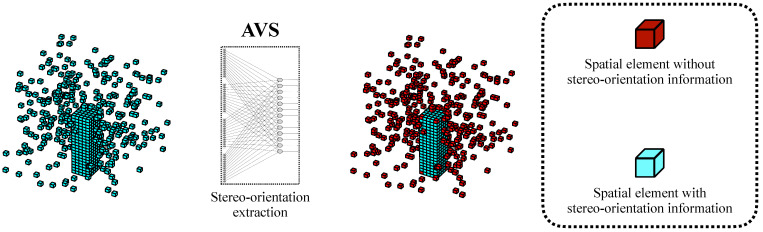
The visualization of information processing within AVS.

We further extended the application of AVS for 3D object classification tasks. The implemented artificial visual system (AVS) based on the mechanism of stereo-orientation selectivity demonstrates high efficiency and remarkable robustness in orientation recognition tasks. Figure 12 illustrates the visualization of information processing within AVS. The stereo-orientation selective cells exclusively respond to the specific spatial orientation information, thereby the spatial elements like noise or some object elements without local orientation information would not elicit neuron responses. In contrast, deep neural network models consider all spatial information and allocate additional attention to spatial elements without stereo-orientation information, resulting in a significant decline in performance.

**Figure 13:**
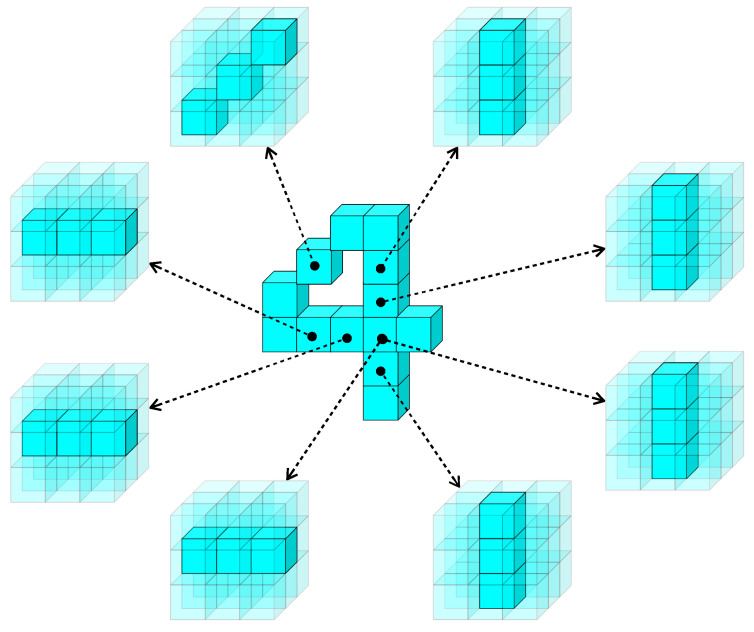
The digital number ‘4’ and its composition of stereo-orientation information.

Similar to our explanation that specific spatial depth information can be organized into a specific stereo-orientation feature, we suggest that complex spatial features are the composition of multiple specific local stereo-orientation information. As illustrated in Figure 13, the digital number ‘4’ can be exactly segmented into eight local stereo-orientation features. Considering the efficiency and robustness of AVS in extracting spatial features, we propose employing AVS as a pre-processing method in 3D object recognition tasks as a potential way to enhance the performance of deep neural network models.

Based on AVS, we initially classify each spatial element as either positive or negative according to the presence or absence of local stereo-orientation information within the local space expanded from each element. The elements identified with orientation information are considered positive, while those without are classified as negative. The details of the separation process for a spatial element in a local space are described in Algorithm 1. Subsequently, we separate the negative and positive information and finally utilize the positive information as input for deep neural network models.
**Algorithm 1:**AVS for feature selection**Require:** A spatial element *p*, local voxel array voxel**Ensure**: 1 if orientation detected in voxels, otherwise 01:**Initialize**: 13 types of local stereo-orientation selective $cell_{i}$2:outs_arr←[0,0,⋯,0]    # length: 133:$\theta \leftarrow 10^{4}$    # cell response sensitivity4:k←2.5    # cell activation threshold5:activation←0    # initial cell activation value6:**for** i=0 **to** 12 **do**7:   $cell_{i}\leftarrow orientation\;selective\;cell$    # create corresponding instance of cell class8:   $activation\leftarrow cell_{i}\left(\theta ,k,voxel\right)$    # cell activation computation9:   outs_arr[i]←activation    #cell activation value: 1 or 010:**end for**11:**if** $\sum_{i=0}^{12}outs\_arr\left[i\right]>0$ **then**12:   **return** 1    #positive information, preserve spatial element *p*13:**else**14:   **return** 0    #negative information, discard spatial element *p*15:**end if**

**Figure 14:**
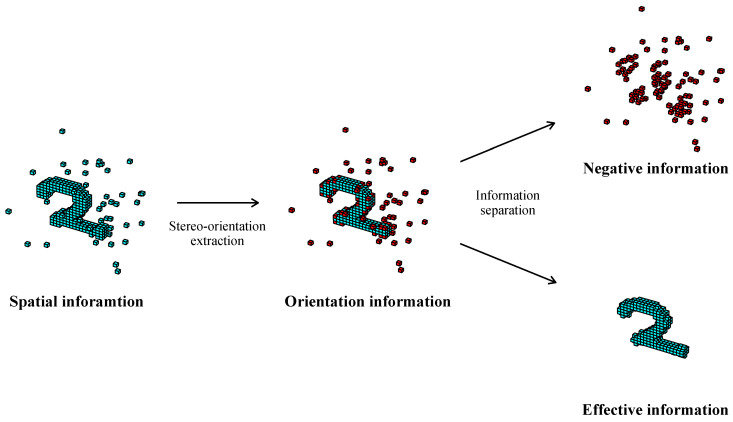
The separation of effective and negative information from original spatial information.

The separation of effective and negative information from original spatial information is illustrated in Figure 14.

The reliability of AVS in recognizing effective object information and enhancing the deep model’s stability and robustness is evaluated on 3D-MNIST and 3D-Fashion-MNIST datasets. TransNeXt and PointMLP, the two models performed well on orientation detection task, were employed as the baseline, and AVS-TransNeXt and AVS-PointMLP were implemented as comparisons to evaluate AVS’s impact on deep model performance and robustness against noise. The models were trained on clean data and evaluated on both clean and noise test sets. The experiments were repeated 3 times under different random seed situations (see Table A2 in Appendix A for more details of training settings). The summarized results are presented in Table 2 and Table 3.

Results Analysis:

- TransNeXt model performed well on clean data but with very poor stability to noise data. The results on noise data (68.3% ± 16.3 and 50.2% ± 10.6) exhibited large performance fluctuation. It is worth noting that anomalous situations occurred in different random seed settings, indicating that TransNeXt is sensitive to the initial parameters and susceptible to noise.
- AVS-TransNeXt model’s performance on clean data is similar to the original TransNeXt. Concurrently, AVS-TransNeXt achieved remarkable noise robustness with small performance fluctuation (97.2% ± 0.2 and 84.4% ± 0.8).
- PointMLP model showed sensitivity and poor stability to noise data. Consequently, its performance was severely compromised, resulting in an accuracy of approximately 12% in 10-classification tasks.
- AVS-PointMLP model demonstrated 2% improved accuracy on clean data compared to the original PointMLP and significant improvement in accuracy and stability on noise data (85.9% ± 1.0 and 75.5% ± 0.3).

In summary, AVS significantly enhanced the baseline models’ robustness and stability on noise data without performance degradation on clean data. AVS is demonstrated as an efficient feature preprocessing system that helps enhance the overall performance of deep models in 3D recognition tasks with minimal computation cost.

### 3.5. Ablation Study

**Figure 15:**
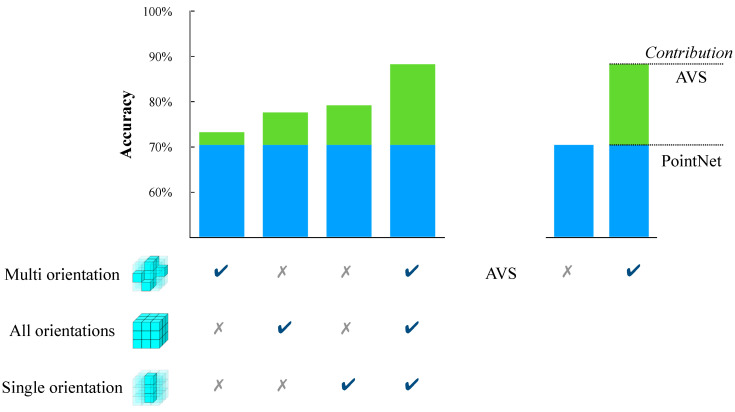
Ablation study on the feature selection process based on local stereo-orientation.

To investigate the contribution of each component in AVS to deep model performance enhancement, we conducted an ablation study. When AVS was employed to enhance deep models’ performance, the complex stereo-orientation selective cells were removed, and the depth selective cells were only responsible for accepting inputs. Accordingly, only the simple stereo-orientation selective cells performed computation in the information separation process. We can modify the separation rules by employing different combinations of simple stereo-orientation selective cells. We evaluated the impacts of different information separation rules based on local stereo-orientation on model performance. Based on the original separation rule, we defined three types of separation rules for positive information: multi-orientation, spatial elements with multiple types of local orientation information but not all; all orientation, the spatial elements with all types of local orientation information; single orientation, spatial elements with only one type of local orientation information. PointNet was employed as the baseline model. Employing the AVS with different separation rules to enhance the baseline model’s performance and record the results as illustrated in Figure 15.

Because different types of information separation rules decided the amount of information extracted from original data, the three methods made different contributions to model performance enhancement. The ’single orientation’ method demonstrated the most significant improvement in model performance, indicating that this method extracted more object features. And when considering all oreintation information states in local space, AVS could significantly improve the model’s performance, suggesting that all simple stereo-orientation selective cells in AVS made a contribution.

## 4. Discussion

Following the thought of local-to-global information aggregation in the Hubel-Wiesel model, we proposed a cascade system to quantitatively explain the generation mechanism of stereo-orientation selectivity and further innovatively developed an artificial visual system for object stereo-orientation selection. Based on the results of physiological visual simulation and object stereo-orientation detection, the proposed mechanism is feasible and effective. The AVS exhibited superiority on orientation extraction tasks compared with deep models. Despite their remarkable learning capability, deep models can learn enough knowledge to complete the detection task with clean data, but they were also affected by other potential spatial features, leading to weak attention to noise. The results demonstrate the weakness of deep models in robustness, which exhibits the gaps between the deep model and the real brain. The extended application of AVS in 3D object classification demonstrated that it can contribute to removing such a gap. After separating the spatial information into effective information (save) and negative information (discard) by AVS, the deep model significantly improved the robustness and stability.

**Figure 16:**
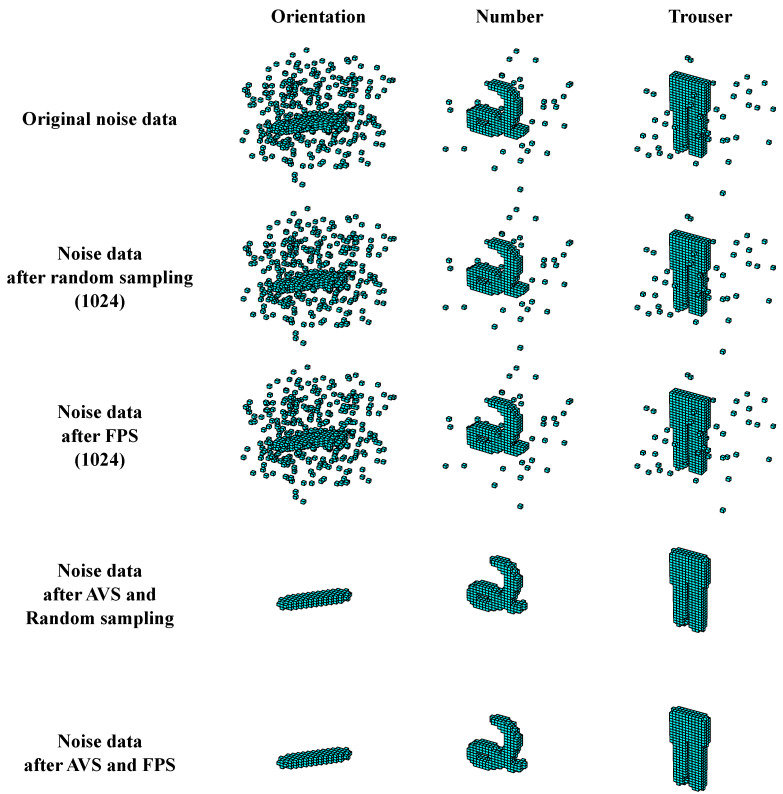
The point sampling effects with and without AVS processing.

It is noteworthy that the deep models employed for 3D object recognition tasks commonly involve a sampling process [96] like random sampling and farthest point sampling (FPS), which often sampling affects model performance. However, conventional sampling methods fail to eliminate the inclusion of noise information during the process. In voxel space, spatial elements of a 3D object are generally clustered together and have a higher probability of forming specific local stereo-orientation fragments, while background noise remains dissociated and independent. Therefore, we can address this issue by employing the AVS to separate noise information and object information. The spatial elements with local stereo-orientation information are identified as effective object information, while the spatial elements without local stereo-orientation information are noise and discarded. Figure 16 illustrates the point sampling effects with and without AVS processing. Notably, under noise situations, the random sampling and FPS methods sampled noise and object information simultaneously, leading to the model performance degradation in the subsequent learning phase. This is because the sampling methods are based on random selection or point coordinates. Consequently, the noise and object information have the same sampling probability under these sampling rules. After incorporating AVS processing, both sampling methods could obtain ideal sampling results, and all noise information was eliminated. Therefore, deep model performance enhancements described above benefit from the AVS completing the noise filtering to reduce the probability of sampling negative information.

We need to state that the current separation rule between negative and effective information remains incomplete. The variations in local stereo-orientation information content also decide the significance of spatial elements for the object recognition process. Furthermore, although AVS processing can effectively eliminate noise information, the subsequent sampling process still includes part of the stochastic process, and object information may be omitted. We can improve the feature extraction ability of AVS through flowing aspects:

- Incorporating the color perception mechanism into AVS to improve performance and generalizability in more complex scenes.
- Introducing a deep learning method to refine the information separation process and reduce information loss due to human design.
- Extending the application of AVS as a feature-based sampling method to avoid information loss during a stochastic process.

In future research, the enhanced AVS is anticipated to be utilized in medical image analysis, geological modeling, robotic vision, and industrial inspection. Furthermore, its fusion performance with other modalities, such as LiDAR and RGB images, will be explored.

## 5. Conclusions

In this paper, we innovatively explained the neural mechanism underlying stereo-orientation selectivity based on the Hubel-Wiesel Model, wherein the generation of stereo-orientation selectivity originates from the convergence and integration of multiple specific depth cues. We further developed an artificial visual system (AVS) for stereo-orientation recognition, comprising simple stereo-orientation selective cells that extract local spatial information and complex stereo-orientation selective cells responsible for integrating local orientation information to exhibit global selectivity. Simulation results demonstrate the effectiveness of AVS in feature extraction while maintaining robustness against spatial noise. Furthermore, we initially explore the integration of AVS with deep neural network models in 3D recognition tasks, which leads to significant enhancements in model robustness, performance, and stability. In conclusion, our research provides a reliable approach for feature extraction 3D object recognition tasks and helps explain how depth information is processed and integrated within the visual system to enable stereo vision.

## Figures and Tables

**Table 1:** Detection accuracy on object orientation datasets.

Method	Noise Intensity						#Prams.	FLOPs
	**0%**	**1%**	**2%**	**3%**	**4%**	**5%**		
ConvNeXt [90]	99.96%	34.83%	26.52%	23.58%	21.65%	21.10%	87.61 M	3890 M
UniRepLKNet [91]	99.04%	19.90%	17.54%	15.98%	16.29%	14.60%	62.35 M	60 M
SwinV2 [92]	95.25%	23.08%	20.94%	20.27%	19.88%	19.34%	49.02 M	210 M
TransNeXt [93]	**100%**	36.71%	34.71%	30.92%	28.63%	25.63%	12.44 M	2630 M
PointNet [94]	99.85%	7.62%	7.69%	7.69%	7.69%	7.69%	3.46 M	230 M
PointMLP [95]	**100%**	8.25%	7.58%	7.58%	7.65%	7.67%	13.23 M	7890 M
AVS	**100%**	**99.71%**	**98.31%**	**96.98%**	**94.98%**	**92.73%**	353	9.48 M

**Table 2:** Detection accuracy of TransNeXt on 3D-MNIST and 3D-Fashion-MNIST datasets.

Dataset	Method	Clean	Noise	Overall
		[%]	[%]	[%]
MNIST	TransNext	**97.6 ± 0.1**	68.3 ± 16.3	73.1 ± 13.6
	AVS-TransNext	97.5 ± 0.2	**97.2 ± 0.2**	**97.2 ± 0.2**
Fashion-MNIST	TransNext	85.5 ± **0.4**	50.2 ± 10.6	56.1 ± 8.9
	AVS-TransNext	**85.7** ± 0.5	**84.4 ± 0.8**	**84.6 ± 0.8**

**Table 3:** Detection accuracy of PointMLP on 3D-MNIST and 3D-Fashion-MNIST datasets.

Dataset	Method	Clean	Noise	Overall
		[%]	[%]	[%]
MNIST	PointMLP	87.8 ± 1.3	12.6 ± **0.7**	25.1 ± **0.7**
	AVS-PointMLP	**91.0 ± 0.4**	**85.9** ± 1.0	**85.7** ± 0.9
Fashion-MNIST	PointMLP	73.7 ± 1.0	13.8 ± 1.2	23.8 ± 1.1
	AVS-PointMLP	**75.9 ± 0.1**	**75.5 ± 0.3**	**75.6 ± 0.3**

## Data Availability

The data presented in this study are available on request from the corresponding author. The data are not publicly available due to data privacy regulations.

## Abbreviations

The following abbreviations are used in this manuscript:

AVS	Artificial Vsiaul System
FPS	Farthest Point Sampling

## Appendix A. Implementation Details of Deep Models

**Table A1:** Training settings for deep models on stereo-orientation recognition tasks.

(Pre-)Training Config	ConvNeXt-Base	UniRepLKNet-N	SwinV2-S	TransNeXt-Micro	PointNet	PointMLP
loss function	cross entropy	cross entropy	cross entropy	cross entropy	cross entropy	cross entropy
optimizer	AdamW	AdamW	AdamW	AdamW	AdamW	AdamW
base learning rate	$1\times 10^{-4}$	$1\times 10^{-3}$	$1\times 10^{-4}$	$1\times 10^{-4}$	$1\times 10^{-4}$	$1\times 10^{-4}$
weight decay	$1\times 10^{-4}$	$1\times 10^{-4}$	$1\times 10^{-4}$	$1\times 10^{-4}$	$1\times 10^{-4}$	$1\times 10^{-4}$
batch size	32	32	32	32	32	32
training epochs	30	30	30	10	30	10
learning rate schedule	exponential decay (0.8)	exponential decay (0.8)	exponential decay (0.8)	exponential decay (0.8)	exponential decay (0.8)	exponential decay (0.8)
number of sampling points	-	-	-	-	512 (random)	512 (FPS)
random seed	42	42	42	42	42	42

**Table A2:** Training settings for TransNeXt, PointMLP, and PointNet on 3D object classification tasks.

(Pre-)Training Config	TransNeXt-Micro	PointMLP	PointNet
loss function	cross entropy	cross entropy	cross entropy
optimizer	AdamW	AdamW	AdamW
base learning rate	$1\times 10^{-5}$	$1\times 10^{-4}$	$1\times 10^{-4}$
weight decay	$1\times 10^{-4}$	$1\times 10^{-4}$	$1\times 10^{-4}$
batch size	32	32	256
training epochs	10	10	30
learning rate schedule	exponential decay (0.8)	exponential decay (0.8)	exponential decay (0.8)
number of sampling points	-	1024 (FPS)	1024 (FPS)
random seeds	[7, 8, 42]	[7, 8, 42]	[7, 8, 42]
